# The function of TLR2 during staphylococcal diseases

**DOI:** 10.3389/fcimb.2012.00167

**Published:** 2013-01-04

**Authors:** Bénédicte Fournier

**Affiliations:** Epithelial Pathobiology Research Unit, Department of Pathology and Laboratory Medicine, Emory University School of MedicineAtlanta, GA, USA

**Keywords:** TLR2, ligands, phagocytosis, neutrophil, *Staphylococcus aureus*

## Abstract

*Staphylococcus aureus* is a versatile pathogen causing a wide range of infections. It has been a major threat both in hospitals and in the community for decades. *S. aureus* is a pyogenic bacterium that elicits recruitment of polymorphonuclear leukocytes (neutrophils) to the site of infection. Neutrophils are among the first immune cells to migrate to an infection site attracted by chemoattractant gradients, usually initiated in response to inflammation. Neutrophil recruitment to an inflammation and/or infection site is a sophisticated process involving their interaction with endothelial and epithelial cells through adhesion molecules. Phagocytes have various receptors to detect pathogens, and they include Toll-like receptors (TLRs). TLRs have been extensively studied over the last 10 years and it is now established that they are critical during bacterial infections. However, the function of TLRs, and more particularly TLR2, during staphylococcal infections is still debated. In this review we will consider recent findings concerning the staphylococcal ligands sensed by TLR2 and more specifically the role of staphylococcal lipoproteins in TLR2 recognition. A new concept to emerge in recent years is that staphylococcal components must be phagocytosed and digested in the phagosome to be efficiently detected by the TLR2 of professional phagocytes. Neutrophils are an essential part of the immune response to staphylococcal infections, and in the second part of this review we will therefore describe the role of TLR2 in PMN recruitment in response to staphylococcal infections.

## Introduction

*Staphylococcus aureus* is a commensal, especially well-adapted to humans, also able to cause a wide range of infectious diseases from minor cutaneous infections to more severe infections such as sepsis, pneumonia, osteomyelitis, and endocarditis. It also frequently infects surgical wounds and medical implants [reviewed in Lowy (1998)]. *S. aureus* has become increasingly resistant to antibiotics in recent years and about 55% of healthcare-associated (formerly hospital-acquired) isolates are now resistant to methicillin (methicillin-resistant *S. aureus*, MRSA) [reviewed in Calfee (2012) and Gould et al. (2012)]. Mostly found in hospitals 20 years ago, MRSA strains have now spread to the community with virulent strains causing necrotizing pneumonia and other severe infections (David and Daum, 2010; Otter and French, 2011). Commensal *S. aureus* is found mainly in the anterior nares and about one third of the populations are permanent asymptomatic carriers. *S. aureus* may also colonize skin, perineum, gastrointestinal tract, and throat [reviewed in Tong et al. (2012)]. *S. aureus* has a formidable repertoire of virulence factors: cell-surface proteins (including protein A) and numerous secreted virulence factors (for example proteolytic enzymes and cytotoxins) contribute to the development of infections by promoting bacterial adhesion, circumventing host immune defenses and causing cell or tissue damage [reviewed in Projan and Novick (1997), Foster (2005), Ben Zakour et al. (2008), Otto (2010), Cheng et al. (2011), and Edwards and Massey (2011)]. Virulence factors are crucial for development of staphylococcal infections; however, host-related determinants, such as immune competence, also play an essential role in infection (Tong et al., 2012). *S. aureus* induces a significant recruitment of leucocytes. Phagocytes, like most immune cells, use pattern recognition receptors (PRRs) to detect and differentiate microorganisms from self. These receptors include the Toll-like receptors (TLRs) which are transmembrane proteins capable of recognizing conserved structures of pathogens, the so-called pathogen-associated molecular patterns (PAMPs). When these pathogen molecules bind to TLRs, a cascade of protein activation results in the activation of NF-κB and activator protein 1, transcriptional factors that promote cytokine production [reviewed in Takeda and Akira (2004) and Oliveira-Nascimento et al. (2012)]. One of the best-characterized TLRs is TLR2 which initiates responses against a wide range of ligands (Ozinsky et al., 2000; Takeuchi et al., 2001, 2002; Sandor et al., 2003). The literature contains diverse descriptions of the function of TLRs during staphylococcal infections. We will review the most recent findings concerning the staphylococcal ligands detected by TLR2 and the role of phagocytosis in TLR2 sensing. We will also consider what is known about the contribution of TLR2 to the recruitment of neutrophils during staphylococcal infections.

## Role of lipoproteins in TLR2-mediated sensing of *S. aureus*

### What do we know about staphylococcal TLR2 ligands?

**Figure 1:**
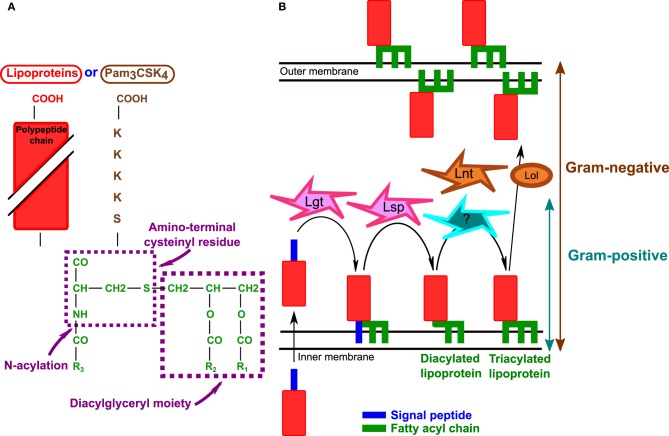
**Bacterial lipoproteins. (A)** Structure of bacterial lipoproteins according to Braun and Wu (1994). The structure of synthetic lipopeptide Pam_3_CSK_4_ is also indicated. Triacylated bacterial lipoproteins and Pam_3_CSK_4_ have the same lipid moiety (green). R_1_, R_2_, and R_3_ are fatty acyl chains. Adapted from Braun and Wu (1994) and Chambaud et al. (1999). **(B)** Biosynthesis of lipoproteins in Gram-negative and Gram-positive bacteria. After crossing the cytoplasmic membrane Lgt (prolipoprotein diacylglyceryl transferase) transfers a diacylglyceride to the polypeptide chain and Lsp (lipoprotein signal peptidase) cleaves the signal peptide. In Gram-negative bacteria Lnt (lipoprotein N-acyl transferase) adds a third fatty acyl chain as indicated in the text. Although triacylated lipoproteins have been described in *S. aureus*, the mechanism of lipoprotein N-acylation is not known. Furthermore, in Gram-negative bacteria, acylated lipoproteins are translocated to the outer membrane by the Lol system [Reviewed in Okuda and Tokuda (2011)].

TLR2 can recognize ligands with very diverse structures such as lipoproteins/lipopeptides, peptidoglycan, glycopolymers [lipoteichoic acid (LTA) and lipoarabinomannan], and proteins (porins, virus capsids) [reviewed in Zahringer et al. (2008) and Oliveira-Nascimento et al. (2012)]. The wide range of TLR2 ligands is partly due to its heterodimerization with co-receptors described to expand the spectrum of stimuli recognized by TLR2 without altering the signaling pathways used (Farhat et al., 2008). TLR2 associated with TLR1 senses triacyl lipopeptides/proteins (represented by the synthetic triacylated lipopeptide, Pam3-Cys-Ser-Lys4, Pam_3_CSK_4_) (Figure 1A) whereas an association with TLR6 is required to recognize diacyl lipoproteins (Pam_2_CSK_4_ or MALP2) (Ozinsky et al., 2000; Takeuchi et al., 2001, 2002; Takeda et al., 2002). Binding of Pam_3_CSK_4_ induces a conformational change of the ectodomains of both TLR1 and TLR2, resulting in the formation of a heterodimer. Pam_2_CSK_4_ does not promote this conformation and therefore does not activate TLR2-TLR1 (Jin et al., 2007). TLR2 may detect other staphylococcal PAMPs: peptidoglycan, a large polymer constituting the main component of the Gram-positive cell wall, and LTA (the counterpart of LPS in Gram-positive bacteria) [reviewed in Fournier and Philpott (2005)] although the relevance is controversial as the concentrations of peptidoglycan and LTA necessary to trigger TLR2 are not physiological (Zahringer et al., 2008). Indeed TLR2 recognition of peptidoglycan has been debated [(Travassos et al., 2004) and reviewed in Fournier and Philpott (2005)]. It has been suggested that muramyl dipeptide, the smallest peptidoglycan structure, is detected by an intracellular PRR, Nod2 (nucleotide-binding oligomerization domain protein 2) (Girardin et al., 2003). Interestingly, it was recently shown that peptidoglycan co-localizes with both TLR2 and Nod2 after internalization. Nevertheless, the interaction of peptidoglycan with TLR2 is not dependent on Nod2 and vice-versa (Muller-Anstett et al., 2010). Furthermore, peptidoglycan potentializes the activity of other TLR ligands, including LTA and LPS (Volz et al., 2010). Thus, it is possible that peptidoglycan is a weak TLR2 ligand that contributes to host immune responses by enhancing inflammation triggered by other more potent staphylococcal ligands such as lipoproteins. In addition to TLR1 and TLR6, other accessory non-TLR molecules may also serve as TLR2 co-receptors [reviewed in Oliveira-Nascimento et al. (2012)]. CD36, an integral membrane scavenger receptor, appears to have a role in LTA recognition and *S. aureus* phagocytosis (Hoebe et al., 2005; Stuart et al., 2005; Triantafilou et al., 2006; Baranova et al., 2008; Nilsen et al., 2008) although it is not clear how important this receptor is for the immune response against *S. aureus* (Baranova et al., 2008). CD36-deficient mice present an increased susceptibility to *S. aureus* and various TLR2 ligands including LTA and MALP2 (Hoebe et al., 2005). In contrast to other TLR2 accessory molecules, mannose-binding lectin (MBL) is a soluble humoral PRR that binds to the carbohydrate moieties of microorganisms in an EDTA-specific manner and in the case of *S. aureus*, LTA [reviewed in Ip et al. (2009)]. MBL-deficient mice are more susceptible than wild-type mice to *S. aureus* septicemia (Shi et al., 2004) and MBL has been reported to enhance *S. aureus* phagocytosis (Neth et al., 2002; Krarup et al., 2005; Ip et al., 2008). Indeed, the serum level of a wide array of cytokines (TNFα, RANTES, MIP-2, MCP-1, KC, IFNγ) is decreased in MBL-deficient mice compared to wild-type mice following *S. aureus* intravenous challenge (Ip et al., 2008). CD36 and MBL both bind to LTA but they interact independently with TLR2 (Ip et al., 2008).

Bacterial lipoproteins were first found to activate TLR2 in 1999 (Aliprantis et al., 1999) and are among the most potent staphylococcal components known to activate TLR2 (Hashimoto et al., 2006a,b). There is increasing evidence that lipoproteins play an important role in TLR2 activation by staphylococci. Lipoproteins of Gram-negative bacteria are sorted to the inner and outer membranes separated by the periplasm and are also found in the outer leaflet of the outer membrane (Figure 1B) (Okuda and Tokuda, 2011). In contrast, lipoproteins of Gram-positive bacteria are only attached into the outer leaflet of the plasma membrane and are present in a subcellular space comprised between the plasma membrane and the peptidoglycan [reviewed in Hutchings et al. (2009)]. Lipoproteins are very abundant in bacteria, and make up about 2–3% of the bacterial proteome [reviewed in Hutchings et al. (2009)]. The typical structure of Gram-positive lipoprotein was determined in Braun and Wu (1994) (Figure 1A). Lipoprotein biosynthesis is a multistep process (Figure 1B). The proteins are first exported across the plasma membrane as preproteins; prolipoprotein diacylglyceryl transferase (Lgt) then transfers a diacyl glyceride to the lipobox cysteine residue in the protein signal peptide and lipoprotein signal peptidase (Lsp) cleaves the signal peptide. In Gram-negative bacteria, lipoprotein N-acyl transferase (Lnt) adds a third fatty acyl chain to the same lipobox, thereby generating a triacylated lipoprotein [reviewed in Hutchings et al. (2009)] (Figure 1B). As Gram-positive bacteria do not possess the *lnt* gene, it was presumed that they contained mainly diacylated lipoproteins. However, recent data suggest that triacylated lipoproteins are also present in *S. aureus*: indeed staphylococcal lipoprotein SitC has three fatty acids (Kurokawa et al., 2009). SitC-stimulated macrophages produce TNFα and IL-6 in a TLR2- and MyD88-dependent but TLR1- and TLR6-independent manner. However, SitC is not as potent as Pam_3_CSK_4_ (Kurokawa et al., 2009). Interestingly, *sitC*^−^ strains can still activate TLR2, implicating other staphylococcal ligands including other lipoproteins in TLR2 activation (Kurokawa et al., 2009). Both diacylated and triacylated lipoproteins have been described in *S. aureus* (Tawaratsumida et al., 2009; Asanuma et al., 2011). A very recent study reported that lipoprotein production is modulated by environmental conditions and this may explain why both diacylated and triacylated lipropoteins have been described in *S. aureus*. Indeed, diacylated forms of lipoproteins are found mainly when the bacteria are in the stationary growth phase or at acidic pH indicating that growth conditions determine lipoprotein acylation in staphylococci (Kurokawa et al., 2012).

An *lgt*-deficient *S. aureus* mutant displays membrane-anchored lipoproteins without the lipid groups, and the full lipoproteins are detected mainly in the culture supernatant (Stoll et al., 2005). The TLR2-dependent production of cytokines (TNFα, IL-10, IL-6, IL-8, and MIP2) in various cell types (mononuclear, epithelial, and endothelial cells) in response to this mutant was very much lower than that in response to the wild type (Stoll et al., 2005; Schmaler et al., 2009; Kang et al., 2011). However, *in vivo* studies with a murine model of septicemia are contradictory: in one study, the *S. aureus lgt*^−^ mutant was more virulent than the WT strain (Bubeck Wardenburg et al., 2006), whereas another study reports lower virulence for this mutant (Schmaler et al., 2009). These various findings nevertheless indicate that staphylococcal lipoproteins are critical for the innate immune response through TLR2. However, the exact role of lipoproteins *in vivo*, and whether lipoproteins are indeed necessary for staphylococcal infections, remain unclear.

### Role of phagocytosis in TLR2 sensing

Phagocytosis of pathogens by professional phagocytes is a key step in innate immunity. Phagocyte receptors (Fc receptors, complement receptors, integrins, scavenger receptors among others) recognize bacteria opsonized with immunoglobulins, complement, or extracellular matrices e.g., fibronectin [Reviewed in Underhill and Ozinsky (2002)]. After major rearrangements of the actin cytoskeleton, phagocytes internalize bacteria (Underhill and Ozinsky, 2002). TLRs are not phagocytic receptors *per se*, but they are also internalized in the process and therefore participate to the link between phagocytosis and inflammatory responses by triggering the production of cytokines (Underhill and Ozinsky, 2002). Engulfed bacteria are transferred to phagosomes, which fuse with other organelles (endosomes and lysosomes) and enzymatic complexes such as NADPH oxidase. The result is a highly hydrolytic phagolysosome with reactive oxygen species that lyses bacteria and digests their components [reviewed in Underhill and Ozinsky (2002), Stuart and Ezekowitz (2005), Kinchen and Ravichandran (2008), and Nordenfelt and Tapper (2011)]. Treatment of THP-1, macrophage-like cells, with live *S. aureus* increases the expression of TLR2 and MyD88 (Zarember and Godowski, 2002). Intriguingly, although MyD88-deficient macrophages release much less cytokines than wild-type macrophages after staphylococcal infection, TLR2-deficient macrophages produce a significant amounts of cytokines, suggesting that other receptors associated with MyD88 are involved in *Staphylococcus* sensing (Takeuchi et al., 2000; Lembo et al., 2003). In 1999, a first study reported that TLRs were recruited to the phagolysosomes where they were capable of differentiating Gram-negative from Gram-positive pathogens to produce cytokines (Underhill et al., 1999). Indeed, dominant-negative inhibitor TLR2 or MyD88 introduced into macrophage-like cells (RAW 264.7) failed to produce TNFα in response to *S. aureus* infection, whereas the wild-type gene did (Underhill et al., 1999). Other studies later suggested that there are two modes of phagosome maturation (constitutive and inducible) and TLRs including TLR2 may accelerate inducible maturation in presence of specific ligands (Blander and Medzhitov, 2004) although this is still controversial (Yates and Russell, 2005).

**Figure 2:**
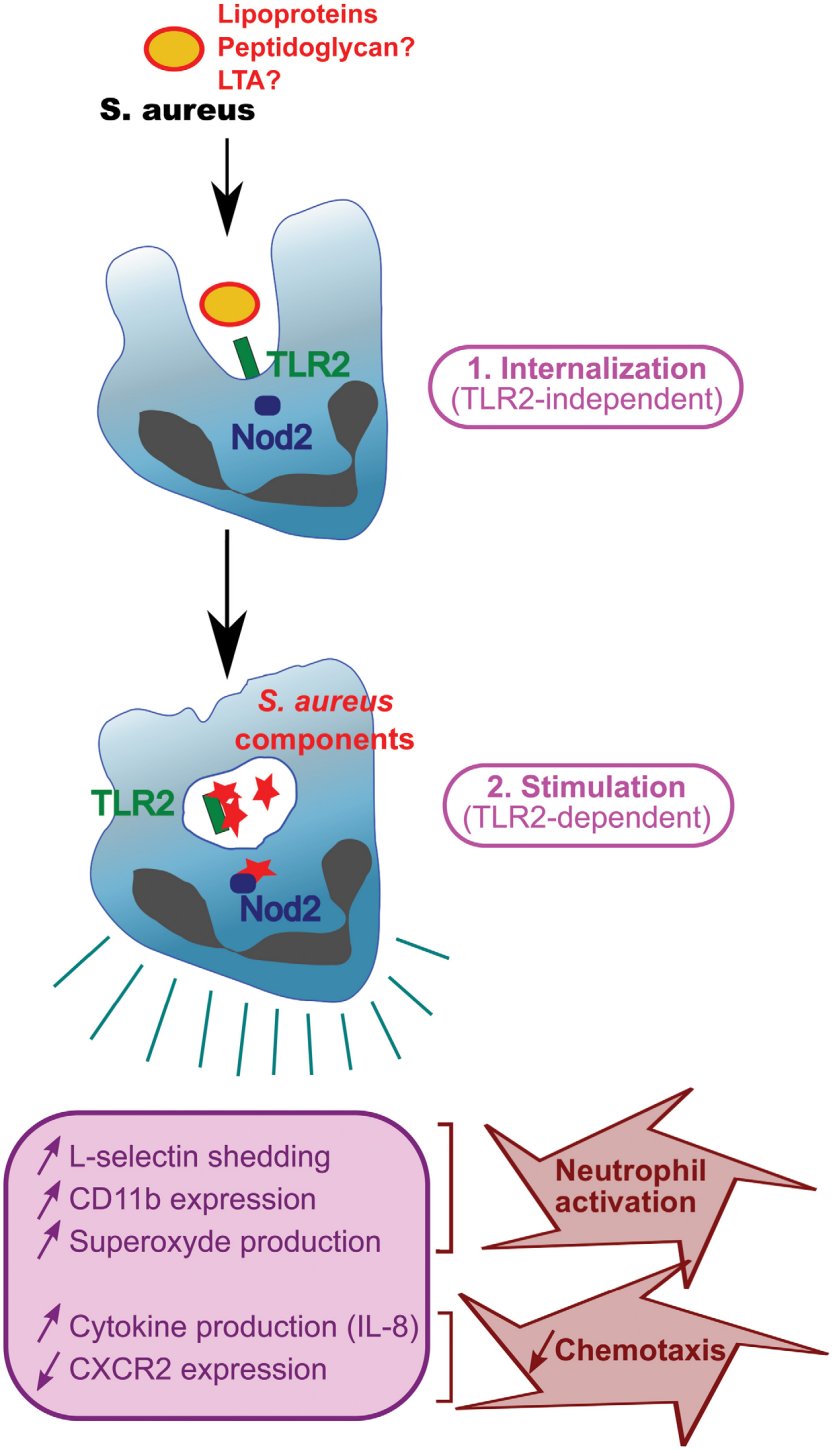
**Function of TLR2 during phagocytosis.** *S. aureus* with surface TLR2 ligands (lipoproteins, peptidoglycan, and LTA) is internalized in a TLR2-independent manner and digested in phagosomes. Staphylococcal components are released and stimulate PRRs e.g., TLR2 or Nod2, resulting in phagocyte activation and production of cytokines.

Several articles demonstrate that staphylococcal degradation in the phagosome is required for PAMP release and recognition by PRRs (Kapetanovic et al., 2007; Dietrich et al., 2010; Ip et al., 2010; Shimada et al., 2010; Wolf et al., 2011). Thus, phagocytosis seems to proceed in two steps. First, bacteria are recognized by TLRs at the surface of the phagocytes and this is not dependent on TLR ligands (Figure 2). Indeed, peptidoglycan from *S. aureus* strains resistant to lysozyme and from wild-type strains release similar amounts of IL-1β and TNFα during the first hour post-infection (Shimada et al., 2010; Wolf et al., 2011), suggesting that peptidoglycan does not require to be degraded at this step. Second, engulfed bacteria are transferred into phagosomes and lysed. During this digestion in the phagosomes, most bacterial cell-wall components and bacterial DNA are released, resulting in materials presented to PRRs (Ip et al., 2010; Wolf et al., 2011). Cytosolic PRRs detecting these structures are either engulfed TLRs such as TLR2, cytosolic TLRs such as TLR9 (a receptor for CpG motifs in DNA), or Nod2 depending on the study (Kapetanovic et al., 2007; Wolf et al., 2011) (Figure 2). Phagocytosis and digestion of pathogens therefore amplify the immune response by presenting motifs recognized by cytosolic PRRs (Wolf et al., 2011). SitC increases intracellular TLR2 and co-localizes with TLR2 (but unlike peptidoglycan, not with Nod2) (Muller et al., 2010; Muller-Anstett et al., 2010). TLR2 and MBL have been reported to co-immunoprecipitate and also co-localize in the phagosome. Importantly, cytochalasin D, a phagocytosis inhibitor, suppresses MBL-induced TNFα, suggesting that internalization is necessary for MLB function (Ip et al., 2008). Thus, internalization seems to be a prerequisite for full *S. aureus*-induced activation of TLR2, at least in professional phagocyte cells.

## TLR2 and neutrophil recruitment during staphylococcal infections

Polymorphonuclear cells (PMN) constitute one of the first lines of defense against pathogens, and are thus an important component of the innate immune response to staphylococcal infections. Neutrophils are equipped with a powerful arsenal of weapons capable of killing most pathogens including *Staphylococcus* [reviewed in Miller and Cho (2011), Fournier and Parkos (2012), and Rigby and Deleo (2012)]. Immune diseases leading to neutropenia or neutrophil dysfunction, such as defects of intracellular killing (chronic granulomatous disease, myeloperoxidase deficiency, and glucose-6-phosphate dehydrogenase deficiency) and degranulation (Chediak-Higashi syndrome), or abnormal chemotaxis (leukocyte adhesion deficiency, Job's syndrome) result in recurrent staphylococcal disease [reviewed in Lakshman and Finn (2001), Spickett (2008), Rigby and Deleo (2012), and Tong et al. (2012)], highlighting the critical function of neutrophils in the immune response against *S. aureus*. Although not directly implicating TLR2, two interesting studies report that children with mutations in MyD88 and IRAK4, a protein kinase involved in TLR signaling (Takeda and Akira, 2004), are at high risk of developing pyogenic bacterial infections, mainly involving Gram-positive bacteria such as staphylococci (Picard et al., 2003; Von Bernuth et al., 2008).

TLR2 is strongly expressed on leukocytes (mainly in peripheral blood, but also spleen) (Zarember and Godowski, 2002) and its expression is increased in GM-CSF- and G-CSF (granulocyte/macrophage colony-stimulating factors)-stimulated neutrophils (Kurt-Jones et al., 2002; Hayashi et al., 2003). TLR2 activation also contributes to PMN stimulation. Indeed, stimulation of human PMN with the TLR2 ligand Pam_3_CSK_4_ induces production of several cytokines (IL-8, MIP-1α, MIP-1β, MIP-3α, and IL-1β) (Hayashi et al., 2003; Sabroe et al., 2003). Activation of TLR2 by Pam_3_CSK_4_ induces L-selectin shedding and increases CD11b expression both involved in PMN migration. Pam_3_CSK_4_ in synergy with the bacteria-derived chemoattractant formyl-methionyl-leucyl-phenylalanine (fMLF) generates superoxide (Figure 2). Interestingly, neutrophils from patients with chronic granulomatous disease presenting a defect in oxidative metabolism do not show increased CD11b and TLR2 expression upon stimulation with Pam_3_CSK_4_ (Kobayashi et al., 2004; Hartl et al., 2008), suggesting that oxidative metabolism is necessary for lipoprotein effect. Furthermore, TLR2 stimulation with Pam_3_CSK_4_ also results in increased neutrophil phagocytosis (Hayashi et al., 2003).

The relevance of TLR2-driven immune responses during staphylococcal infections and more particularly neutrophil recruitment has been recently studied. TLR2 appears to contribute to mouse survival in a model of *Staphylococcus*-induced septicemia, but only against intravenous challenge with 10^7^ bacteria (Takeuchi et al., 2000). In a murine model of staphylococcal cutaneous infection, TLR2^−/−^ mice have larger lesions than wild-type mice although neutrophil recruitment was not impaired on day 1 post-infection (Molne et al., 2000; Miller et al., 2006). Work with murine models of staphylococcal skin infections, arthritis and septicemia has led to the suggestion that IL-1R, that also signals through MyD88, is more important than TLR2 for neutrophil recruitment and bacterial clearance [(Hultgren et al., 2002; Verdrengh et al., 2004; Miller et al., 2006), reviewed in Sethi and Chakraborty (2011)]. Intriguingly, neutrophil recruitment in a model of murine peritonitis is significantly lower in TLR2^−/−^ mice than in wild-type mice after 4 h, whereas no difference was observed after 24 h (Mullaly and Kubes, 2006). In contrast, the difference between wild-type and IL-1R-deficient mice in a model of septicemia appeared only after 72 h (Verdrengh et al., 2004). It is thus possible that TLR2 is involved in the very early immune response, whereas IL-1R may take over for subsequent PMN recruitment.

Polymicrobial infections may complicate matters further. Indeed, staphylococcal infection following a mild or severe systemic inflammatory response syndrome (SIRS) (Muckart and Bhagwanjee, 1997) generates different subsets of neutrophils: normal PMNs are found in untreated mice, whereas PMN-I and -II subsets are detected in mice with mild and severe SIRS, respectively (Tsuda et al., 2004). These subsets of neutrophils differ in their capacity to activate macrophages, produce cytokines and express TLRs, TLR2 mRNA being equally abundant in all the subsets (Tsuda et al., 2004). Neutrophil recruitment is impaired during sepsis, one of the frequent manifestations of staphylococcal disease (Zou et al., 2011; Kovach and Standiford, 2012). Although the mechanisms dysregulating PMN recruitment during sepsis are multifactorial, the massive production of chemokines often contributes to neutrophil desensitization [reviewed in Kovach and Standiford (2012)]. Neutrophil recruitment is a multistep process initiated by the presence of a chemoattractant gradient that guide neutrophils to the site of inflammation [reviewed in Ley et al. (2007)]. Two receptors involved in chemoattractant detection, CXCR2 and CXCR1, are abundantly expressed at the surface of neutrophils and both recognize two neutrophil chemoattractants, CXCL-6 (or granulocyte chemotactic protein-1) and CXCL-8 (also known as IL-8) among other chemokines (Stillie et al., 2009; Stadtmann and Zarbock, 2012). High concentration of chemoattractants during sepsis dysregulates neutrophil sensing by down regulating chemoattractant receptors and activating neutrophils, resulting in impaired recruitment (Phillipson and Kubes, 2011). Indeed, peripheral neutrophils during polymicrobial sepsis exhibit only weak CXCR2 expression (Cummings et al., 1999) due in part to TLR activation by microbial ligands. Lipopeptide-treated neutrophils exhibit decreased expression of CXCR2 and CXCR1 (Hayashi et al., 2003; Sabroe et al., 2003), with the loss of CXCR2 expression being quicker and more substantial than that of CXCR1 (Sabroe et al., 2005). It has also been described that treatment with Pam_3_CSK_4_ and MALP2 diminishes neutrophil chemotaxis toward IL-8 or fMLF (Hayashi et al., 2003; Chin et al., 2009). Thus, decreased expression of IL-8 receptors (CXCR-1 and CXCR-2) associated with enhanced chemoattractant production during sepsis may impair perception of chemoattractants by PMN and thereby contribute to defective PMN chemotaxis (Hayashi et al., 2003) (Figure 2). TLR2 activation by lipopeptides 24 h after a low-dose staphylococcal infection results in lethal sepsis with increased bacterial load and a very large decrease of neutrophil numbers (Navarini et al., 2009). Furthermore, TLR2-deficient mice with acute polymicrobial peritonitis, induced by cecal ligation and puncture, exhibit enhanced neutrophil migration, resulting in a decreased bacterial load and serum cytokine production (Alves-Filho et al., 2009). Thus, TLR2 participates actively to neutrophil stimulation and recruitment during staphylococcal infections.

## Conclusion

Staphylococcal diseases have become a significant health issue, and there is a dearth of appropriate antibiotics to treat them. The role of PRRs, including TLR2, during these infections is thus of particular interest. Our knowledge of the staphylococcal components involved in TLR2 recognition has improved substantially, and lipoproteins have been shown to be among the most potent TLR2 ligands in *S. aureus*. The environment-dependent regulation of lipoprotein acylation necessary for detection by TLR2 complicates the issue, and raises questions about how this regulation may modulate host immune responses *in vivo*. Although there is a consensus concerning the role of phagocyte TLR2, stimulated with staphylococcal ligands *in vitro*, reports on the role of lipoproteins in murine staphylococcal infections *in vivo* are more discordant. Further *in vivo* studies are therefore required to elucidate lipoprotein function in the immune response against *S. aureus*. TLR2 is also a major player in staphylococcal disease through its diverse roles in various professional phagocyte functions: PMN stimulation and adhesion molecule expression, chemotaxis and chemoattractant receptor expression, and phagocytosis and ligand detection (Figure 2).

### Conflict of interest statement

The author declares that the research was conducted in the absence of any commercial or financial relationships that could be construed as a potential conflict of interest.
